# Arginase Inhibition Supports Survival and Differentiation of Neuronal Precursors in Adult Alzheimer’s Disease Mice

**DOI:** 10.3390/ijms21031133

**Published:** 2020-02-08

**Authors:** Baruh Polis, Kolluru D. Srikanth, Vyacheslav Gurevich, Naamah Bloch, Hava Gil-Henn, Abraham O. Samson

**Affiliations:** 1 Drug Discovery Laboratory, The Azrieli Faculty of Medicine, Bar-Ilan University, Safed 1311502, Israel; naamah.bloch@biu.ac.il (N.B.); avraham.samson@biu.ac.il (A.O.S.); 2 Laboratory of Cell Migration and Invasion, The Azrieli Faculty of Medicine, Bar-Ilan University, Safed 1311502, Israel; dutt415@gmail.com (K.D.S.); Hava.Henn@biu.ac.il (H.G.-H.); 3 Laboratory of Cancer Personalized Medicine and Diagnostic Genomics, The Azrieli Faculty of Medicine, Bar-Ilan University, Safed 1311502, Israel; slavagur13@gmail.com

**Keywords:** Alzheimer’s disease, neurogenesis, norvaline, arginase, arginine, NO

## Abstract

Adult neurogenesis is a complex physiological process, which plays a central role in maintaining cognitive functions, and consists of progenitor cell proliferation, newborn cell migration, and cell maturation. Adult neurogenesis is susceptible to alterations under various physiological and pathological conditions. A substantial decay of neurogenesis has been documented in Alzheimer’s disease (AD) patients and animal AD models; however, several treatment strategies can halt any further decline and even induce neurogenesis. Our previous results indicated a potential effect of arginase inhibition, with norvaline, on various aspects of neurogenesis in triple-transgenic mice. To better evaluate this effect, we chronically administered an arginase inhibitor, norvaline, to triple-transgenic and wild-type mice, and applied an advanced immunohistochemistry approach with several biomarkers and bright-field microscopy. Remarkably, we evidenced a significant reduction in the density of neuronal progenitors, which demonstrate a different phenotype in the hippocampi of triple-transgenic mice as compared to wild-type animals. However, norvaline showed no significant effect upon the progenitor cell number and constitution. We demonstrated that norvaline treatment leads to an escalation of the polysialylated neuronal cell adhesion molecule immunopositivity, which suggests an improvement in the newborn neuron survival rate. Additionally, we identified a significant increase in the hippocampal microtubule-associated protein 2 stain intensity. We also explore the molecular mechanisms underlying the effects of norvaline on adult mice neurogenesis and provide insights into their machinery.

## 1. Introduction

The adult murine brain continuously generates neuronal progenitor cells (NPCs) in the subventricular zone (SVZ) and subgranular zone (SGZ) of the hippocampal dentate gyrus [1]. However, the existence of human adult neurogenesis has been a subject of intense scientific debate until recently. Tobin et al. (2019) evidenced hippocampal neurogenesis persisting throughout life in the brains of centenarians and even of Alzheimer’s disease (AD) patients [2]. Convincingly, they demonstrated that the density of NPCs, neuroblasts, and immature neurons significantly decreases in cases of mild cognitive impairment and in clinical AD as compared to healthy controls. 

In addition, various animal models of AD are characterized by diminished adult neurogenesis. In particular, triple-transgenic mice (3 × Tg) show an age-dependent neurogenesis insufficiency that is detectable in the hippocampus starting at four months of age [3]. Of note, the decline of neurogenesis in this AD model precedes the manifestation of classical hallmarks of AD pathology, such as deposition of amyloid plaques and neurofibrillary tangles in the brain, as well as memory impairment. Remarkably, as a form of neuroplasticity, adult neurogenesis has been shown to modulate vulnerability to degenerative processes and influence the course of AD [4]. Moreover, various supporting adult neurogenesis treatment strategies have demonstrated their competence to counteract the pathological behavioral outcomes in murine AD models [5].

Nevertheless, the mechanisms that regulate NPC proliferation, differentiation, and migration remain poorly understood. Several reports have demonstrated the effects of nitric oxide (NO) donors upon the rate of NPC proliferation and migration. Zhang et al. (2001) evidenced a substantial increase in the rate of SGZ neurogenesis following treatment with NO donors in rats [6]. Lu et al. (2003) demonstrated upregulation of neurogenesis and a reduction in functional deficits following the administration of an NO donor after traumatic brain injury in rats. [7]. Chen et al. (2004) proved the potency of a complex therapy for stroke with an NO donor and human bone marrow stromal cells to enhance neurogenesis in rats [8]. In contrast, nitric oxide synthase (NOS) deficiency has been shown to impede neurogenesis. Reif et al. (2004) reported a significant decrease in NPC proliferation in the SGZ of NOS3 knockout mice, accompanied by a decline in the levels of vascular endothelial growth factor (VEGF) [9]. These authors suggest a principal role of NOS3 in the stimulation of neurogenesis. Other groups have shown that the effects of NOS3 on progenitor cells are mediated via VEGF. Chen et al. (2005) showed that NOS3 is a downstream mediator of VEGF [10]. They suggest that NOS3 regulates brain-derived neurotrophic factor (BDNF) expression in the ischemic brain and influences NPC proliferation and migration. A more recent report by Jin et al. (2017) revealed that endogenous neuronal NOS1 positively regulates neurogenesis [11]. These authors demonstrated that NOS1 repression decreases neuronal differentiation, and vice versa, as NOS1 upregulation promotes it. 

The semi-essential amino acid, arginine, is a mutual substrate for both NOS and arginase. Brain arginine deprivation, due to arginase over-activation, has been suggested as a cause of AD [12]. Consequently, arginine supplementation [13] and/or arginase inhibition have been proposed to halt AD development [14], and have been successfully tested in AD mice [15]. Of note, non-proteinogenic unbranched-chain amino acid norvaline was shown to amplify the NO levels and reduce urea production and it has been used successfully to treat artificial metabolic syndrome in rats. Previously, we showed that norvaline upsurges the hippocampal levels of NOS3 [16] and NOS1 [17] in AD model mice. 

Additionally, our advanced proteomics assay revealed that chronic treatment of 3 × Tg mice with norvaline led to the activation of several critical for adult neurogenesis biological processes [15]. One of the most significant pathways detected was the neuregulin (NRG) pathway. Of note, NRGs comprise a cluster of epidermal growth factor-like proteins that are highly involved in neural development and brain homeostasis [18]. Accumulating evidence suggests a strong impact of NRG1 signaling upon cognitive function and neuropathology in AD. The overexpression of NRG1 in the hippocampus of AD mice improves memory and ameliorates disease-associated neuropathology [19]. Moreover, the systemic administration of NRG1 intensifies neurogenesis in the mouse dentate gyrus [20,21]. 

Previously, we demonstrated that norvaline treatment up-regulated the VEGF signaling pathway in 3 × Tg mice [15]. We also noted that NOS3 mediates VEGF activity [10]. Since norvaline elevates NOS3 levels [16], we suggest a significant involvement of VEGF activation in the phenotype observed following norvaline treatment. VEGF is essential for neuroprotection [22], and its activation is beneficial for individuals with early signs of AD-associated dementia [23]. VEGF preconditioning has been shown to attenuate age-related decay of adult hippocampal neurogenesis in mice [24]. Additionally, the administration of VEGF in rats with focal cerebral ischemia reduces the infarct size and enhances neurogenesis [25].

Likewise, we disclosed that norvaline treatment led to a significant increase in the levels of the glial cell-derived neurotrophic factor (GDNF) receptor RET (REarranged during Transfection) [15], which is a common signaling receptor for GDNF-family ligands [26]. Of note, GDNF is down-regulated in 3 × Tg mice [27], and its overexpression improves cognitive function in this AD model [28]. Moreover, GDNF supports neuronal survival [29], and RET is essential for mediating the neuroprotective and neuroregenerative effects of GDNF [30]. This factor has been shown to increase neurogenesis in the adult hippocampus [31]. Furthermore, neural cell adhesion molecule (NCAM), a second signaling receptor of GDNF [32], demonstrated a 43% level elevation following norvaline treatment [15]. Of note, NCAM regulates synaptic plasticity [33] and mediates axonal growth in hippocampal and cortical neurons [32]. Consequently, an upsurge in its hippocampal levels points to the treatment-associated improvement of neuroprotective mechanisms in 3 × Tg mice brains. 

Growing evidence indicates that another neuroprotective factor, nerve growth factor (NGF), plays a crucial role in the pathogenesis of AD [34]. NGF administration was shown to promote neurogenesis in adult rodents [35]. Accordingly, NGF application has emerged as a promising approach in AD therapy [36]. The neuroprotective effects of NGF are mediated via tropomyosin receptor kinase A [37], which demonstrated a significant 56% increase following norvaline treatment [15]. 

Additionally, we disclosed a significant, more than two-fold elevation, in the levels of neuroligin-1 in the hippocampi of 3 × Tg mice following treatment with norvaline [16]. Neuroligin-1 knockdown has been shown to reduce the survival rate of adult-generated newborn hippocampal neurons [38]. This group previously demonstrated that neuroligin-1 overexpression selectively increases the degree of excitatory synapse formation in adult mice [39].

Overall, several lines of converging evidence unequivocally point to the manifold effects of norvaline upon neurogenesis, neuronal cell differentiation, and migration. Consequently, it is plausible to hypothesize that this substance induces adult neurogenesis in AD model mice. In order to check this hypothesis, we applied an innovative immunohistochemistry approach, using several biomarkers and bright-field microscopy, to study the effects of arginase inhibition with norvaline upon the rate of neurogenesis in 3 × Tg mice. We demonstrate that norvaline promotes neuronal differentiation and survival. We also explore the molecular mechanisms underlying the effects of norvaline on adult neurogenesis and provide insights into their machinery.

## 2. Results

### 2.1. 3 × Tg Mice Show a Significantly Reduced DCX-Positive NPC Density, Compared to Wild Type Mice, which Demonstrate a Dissimilar Phenotype, and are Unaffected by Arginase Inhibition 

Typically, in mice, the newborn cells of the SGZ migrate into the granule layer and extend their dendrites into the molecular layer [40]. Immunohistochemical staining for doublecortin (DCX) of coronal serial sections through the dentate gyrus efficiently revealed newly generated cells. 

DCX is a microtubule-associated phosphoprotein, which efficiently labels late mitotic neuronal precursors and early postmitotic cells [41], and is widely used as a reliable marker for newly-born neurons in the adult hippocampus [42]. DCX-positive cells express other early neuronal antigens but are deficient of antigens specific for glia or apoptotic cells [43]. DCX expression is profound in dendrites; accordingly, newly-born neurons’ absolute number and dendritic growth can be efficiently evaluated with DCX immunostaining technique.

**Figure 1:**
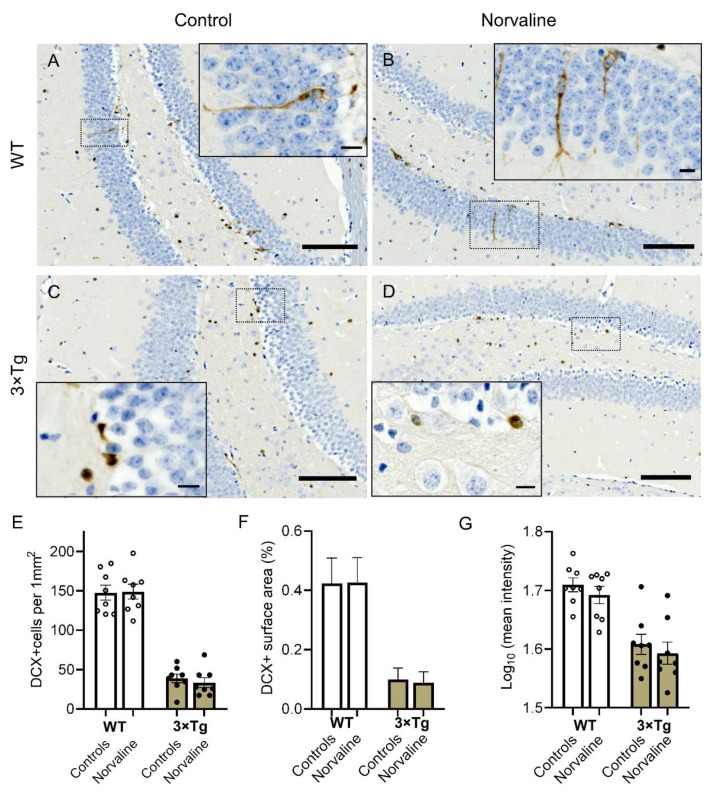
Representative ×40 bright-field micrographs showing the distribution of newly generated doublecortin-positive (DCX+) cells in the dentate gyrus of 7-month-old male wild-type (WT) (**A**,**B**) and 3 × Tg (**C**,**D**) mice in relation to norvaline treatment. Scale bars represent 100 µm. Brain sections were counterstained with hematoxylin. Newborn cells (brown reaction product) are present in both blades of the dentate gyrus and the hilus area. Insets (**A**,**B**) with ×100 magnified views show DCX+ neurons somata in the inner third of the granule cell layer. The cells possess bipolar shape and demonstrate dendritic growth into the outer dentate molecular layer. Insets (**C**,**D**) show spherical-shaped and processes-deficient DCX+ neurons clustering in the SGZ. Scale bars indicate 10 µm. (**E**) DCX+ cell density. (**F**) DCX immunopositive surface area. (**G**) DCX stain intensity. The data are presented as means ± SEM, *n* = 8.

Seven-month-old male wild-type (WT) animals showed characteristic patterns of DCX expression (Figure 1A). The observed DCX-positive cells were distributed heterogeneously within different regions of the dentate gyrus and were arranged in clusters (Figure 1A,B). Remarkably, in the WT, the vast majority of the hippocampal DCX-positive neurons were situated in the SGZ; still, a substantial part of them were visible in the granular layer (Figure 1A,B insets). These bipolar cells demonstrated extensive dendritic growth into the molecular layer.

In contrast, the 3 × Tg mice DCX-positive cells did not exhibit extensive dendrites, and are marginally present in the granular layer (Figure 1C,D). Two-way ANOVA test revealed a significant effect of genotype on DCX positivity with a significant (*p* < 0.0001; F_1, 28_ = 203.2) reduction in the levels of DCX positive surface area (Figure 1F), cell density (Figure 1E), and mean stain intensity (Figure 1G) in 3 × Tg mice as compared to WT age-matched animals. The treatment factor had no significant influence upon these parameters. Additionally, the interaction accounted for less than 0.1% of the total variance. 

### 2.2. Norvaline Caused an Escalation of the PSA-NCAM Levels in the Hippocampi of 3 × Tg Mice, as Evidenced by an Increase in Immunopositive Surface Area and Stain Intensity

**Figure 2:**
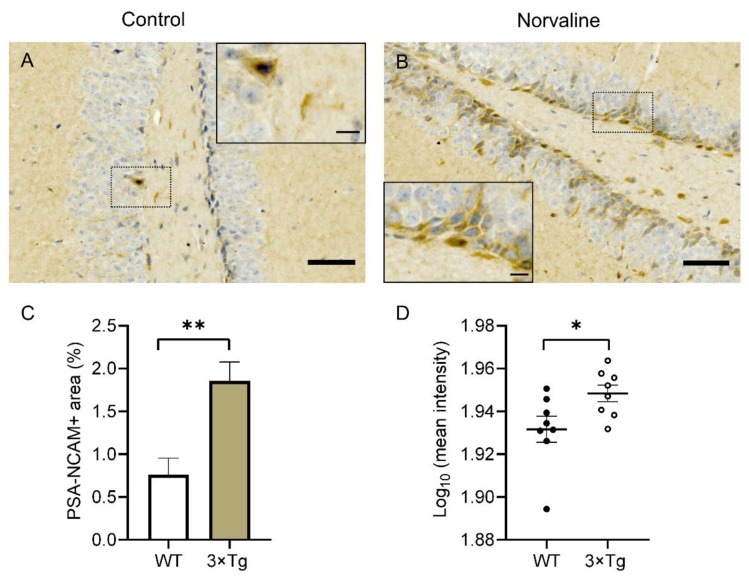
Representative ×40 bright-field micrographs of the hippocampal dentate gyri of 3 × Tg mice with ×100 insets (**A**,**B**). The subgranular zone (SGZ) located polysialylated neuronal cell adhesion molecule (PSA-NCAM) positive cells are marginally present in vehicle-treated animals (**A**) but show much greater incidence in norvaline-treated mice with penetration into the granule cell layer (**B**). The treatment is associated with a significant increase in the PSA-NCAM immunopositive area (**C**) and stain intensity (**D**). Scale bars 50 µm, insets 10 µm. The data are presented as means ± SEM. * *p* < 0.05, ** *p* < 0.01, (two-tailed Student’s *t*-test), *n* = 8.

In order to corroborate the norvaline effects upon the rate of newly generated neurons survival and differentiation rate in adult 3 × Tg mice, we tested the hippocampal levels of polysialylated neuronal cell adhesion molecule (PSA-NCAM) expression via immunohistochemistry. We observed a significant effect of the treatment on PSA-NCAM expression in SGZ, which is characterized by an increase in the levels of stain intensity (Figure 2D) and the immunopositive surface area (from 0.76 ± 0.2% to 1.86 ± 0.22%) (Figure 2C). Of note, PSA-NCAM-positive cells were scarcely present in the SGZ of 3 × Tg mice and did not penetrate the granular layer (Figure 2A). In contrast, these neurons were frequent in the SGZ and the granular layer of the 3 × Tg mice treated with norvaline (Figure 2B).

### 2.3. Norvaline Rescues Neuronal and Dendritic Loss in 3 × Tg Mice, as Evidenced by MAP2 Staining

The dynamic behavior of microtubules is crucial during cell division. Microtubule-associated protein 2 (MAP2) is a neuron-specific protein stabilizing dendritic microtubules; thus, it serves as a reliable neuronal marker [44]. MAP2-positive neurons possess relatively large cell bodies (more than 20 µm in diameter) and one or more dendrites (50 µm or longer) [45]. 

**Figure 3:**
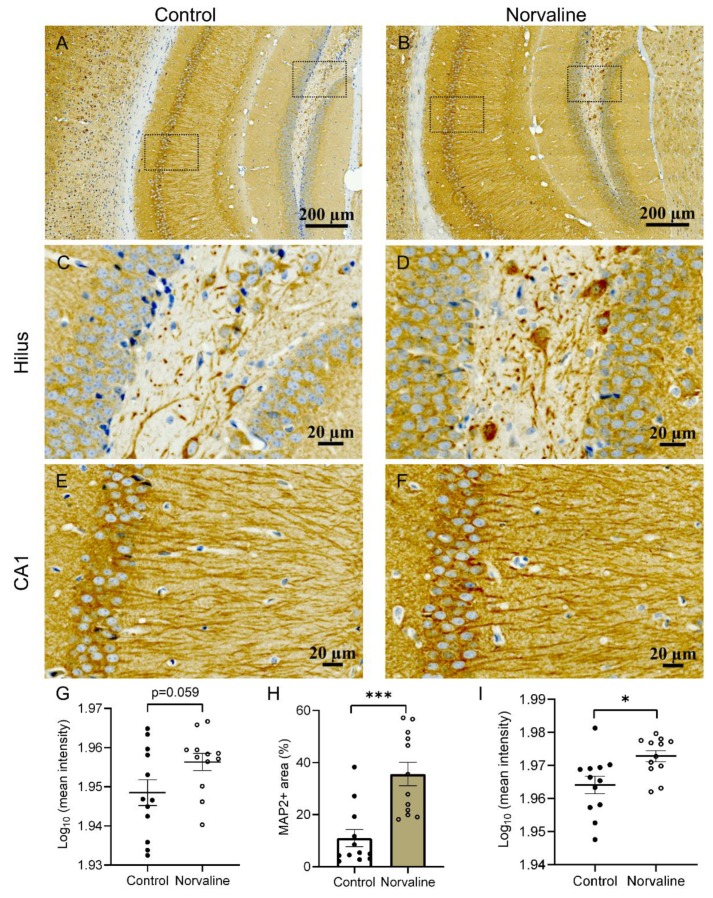
Representative ×20 bright-field micrographs of the 3 × Tg mice hippocampi (**A**,**B**). ×40 magnification of hilus (**C**,**D**) and CA1 region (**E**,**F**). Norvaline treatment led to a significant increase in CA1 MAP2-immunopositive surface area (**H**), and stain intensity (**I**). (**G**) Mean MAP2 stain intensity in hilus area. The data are presented as means ± SEM (*n* = 12, four brains per group, three sections per brain). *** *p* < 0.001, * *p* < 0.05 (two-tailed Student’s *t*-test).

We measured the mean stain intensity of the hippocampal MAP2-positive objects and the immunopositive surface area. MAP2-positive objects were quantified in the *cornu ammonis I* (CAI) (Figure 3E,F) and hilus areas (Figure 3C,D). Norvaline-treated brains demonstrated robust MAP2 signal, while vehicle-treated brains exhibited a decrement in MAP2 signal, as evidenced by two-tailed Student’s t-test. We observed a significant effect of the treatment (*p* = 0.0002, *t* = 4.403, *df* = 22) on MAP2-positive area (with more than three-fold increase) in the CA1 region (Figure 3H). Stain intensity also demonstrated a significant elevation in CA1 (Figure 3I). Analysis of the same parameters in the hilus area did not reveal any significant effect, though stain intensity increased with a *p*-value of 0.059 (Figure 3G). 

### 2.4. Norvaline Escalates the Transcription Levels of C-C Motif Chemokine 11

Eosinophil chemotactic protein or C-C motif chemokine 11 (CCL11) has been shown to promote the migration and proliferation of NPCs in vivo and in vitro [46]. In order to decipher the mechanisms of observed treatment-associated differences in adult neurogenesis, we examined the transcription levels of this β-chemokine in the hippocampi of 3 × Tg mice. 

**Figure 4:**
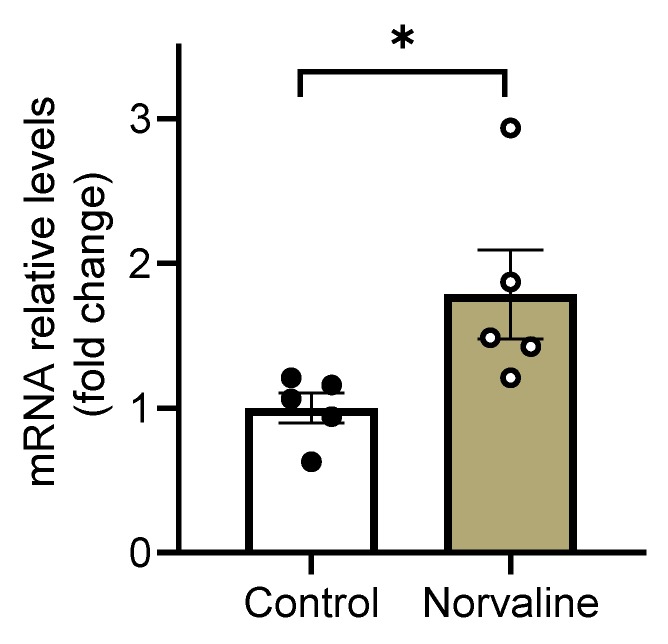
Hippocampal CCL11 mRNA expression levels. Real-time polymerase chain reaction (RT-PCR) analysis of mRNA levels of CCL11 gene. The normalized data are presented as the mean ± SEM (*n* = 5 brains per group). * *p* < 0.05 (two-tailed Student’s *t*-test).

Remarkably, the levels of CCL11 mRNA are 79% higher in the norvaline treated mice than in controls. The Student’s t-test demonstrated the significance of the difference between the means of control and treated animals (*p* = 0.0415, *t* = 2.425, *df* = 8) (Figure 4).

### 2.5. Arginase Inhibition with Norvaline Increases the Hippocampal Expression Levels of PAX6 Protein

Paired box protein (PAX6) postnatal expression is prominent in neurons of different brain regions, including SGZ and SVZ. Nacher et al., (2005) evidenced strong PAX6 expression in proliferating precursors of the adult rodent dentate gyrus. Also, they demonstrated PAX6 expression in nonproliferating cells, which are in early developmental stages [47].

**Figure 5:**
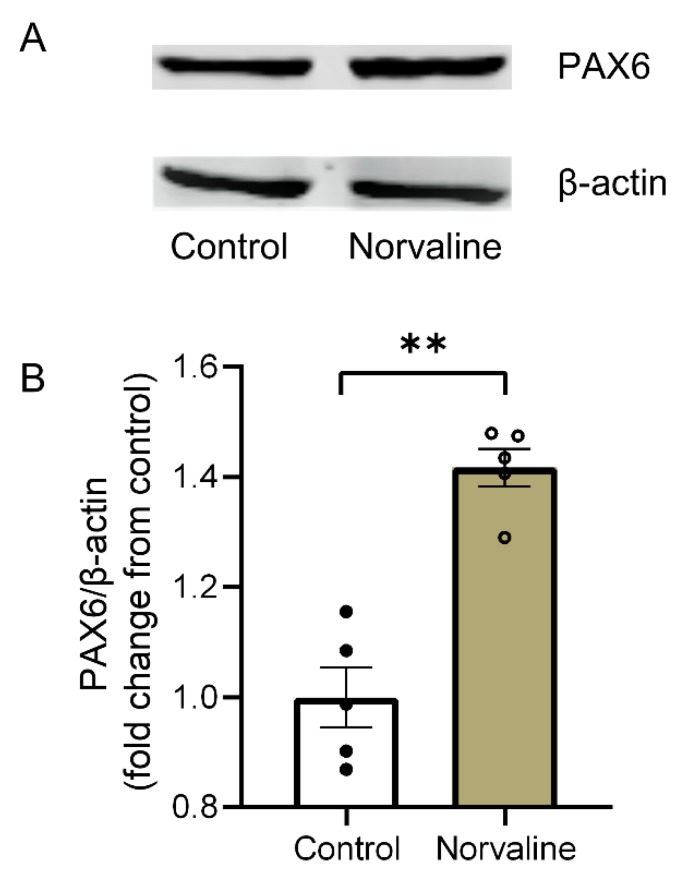
Western blot analysis of the hippocampal lysates using anti-PAX6, and anti-β-actin antibodies (**A**). The normalized data (**B**) are presented as the mean ± SEM (*n* = 5 brains per group). ** *p* < 0.01 (two-tailed Student’s *t*-test).

We applied an immunoblotting technique with a monoclonal anti-PAX6 antibody to study the treatment-associated changes in the hippocampal PAX6 expression levels (Figure 5A) and revealed a significant 42% increase in PAX6 protein levels following the treatment (Figure 5B). 

### 2.6. Norvaline Activates MAPK/ERK Pathway

In order to decipher the biological mechanisms of norvaline effects upon adult neurogenesis in AD mice, we performed advanced microarray analysis with 875 phosphosite-specific antibodies and 451 pan-specific antibodies. This technique is sensitive, convenient, and cost-efficient [48]. We tracked the differential binding of biotin-labeled proteins in lysates prepared from hippocampal tissues and applied the strict criteria to detect proteins and phosphoproteins that demonstrated a significant change in levels following the treatment with norvaline. Priority leads were selected in accordance with the following criteria: %CFC ≥ 75%, sum of errors < 75% of %CFC, and average normalized net signal ≥ 1500. Possible leads were selected with: %CFC ≥ 45% but < 75%, sum of errors < 80% of %CFC, average normalized net signal ≥ 750. This method revealed 48 priority and possible targets (Table S1).

**Figure 6:**
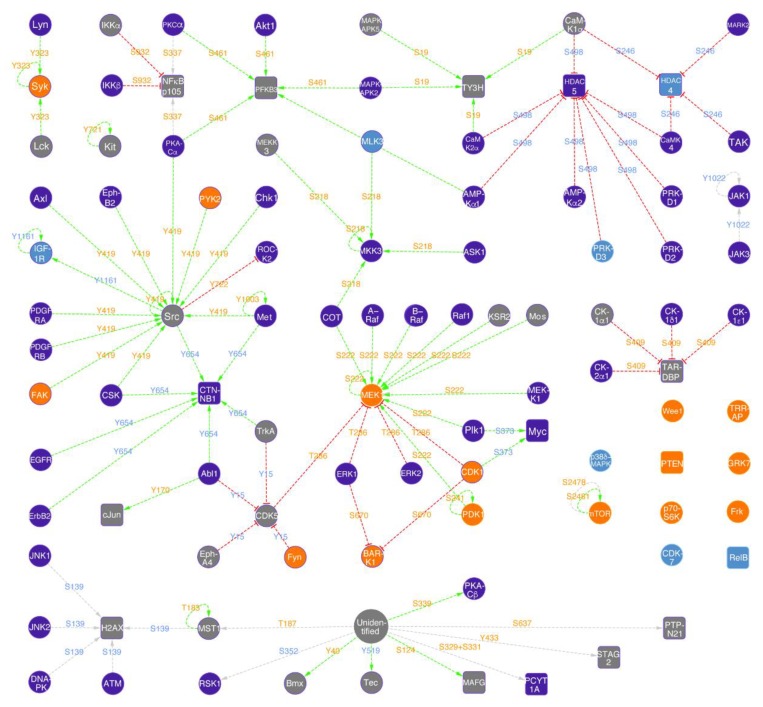
KiNetscape representation of norvaline treatment associated hippocampal signaling (qualitative map style). Protein kinases are represented with circular icons and other proteins with rounded box icons (nodes). Activating phosphorylation events are shown with green dotted lines and arrows, inhibitory phosphorylation with red dotted lines and phosphorylation with undefined effects with grey dotted lines (edges). Proteins that showed increased expression changes greater than 45% are colored orange, and appear blue if there was a decreased expression greater than 45%. Protein expression changes less than 45% are not identified and these protein icons are colored purple. If the phosphorylation of a site on a protein was induced by more than 45%, then the text for this phosphosite is colored orange. If its phosphorylation was reduced more than 45% in response to norvaline, the text is colored blue. Changes in phosphorylation less than 45% are not indicated and the text for these phosphosites appears grey. The appearance of a positive or negative sign in front of the phosphorylation site text indicates if the site is known to be stimulatory or inhibitory, respectively.

The most prominent target discovered was mitogen-activated protein (MAP) kinase MEK1 phosphorylated at serine 222, with the treatment-associated increase in levels by 1495% (Table S1). Moreover, we show a two-fold escalation in MEK1 protein levels, which was disclosed previously by another assay [15]. Of note, another MEK1 phosphorylation site at threonine 286 demonstrated a 446% increase, which indicates a complex regulatory norvaline effect upon the MAPK/ERK pathway (Figure 6). 

In addition, our assay revealed several other neurogenesis-related proteins. For instance, Jun proto-oncogene-encoded AP1 transcription factor phosphorylated at tyrosine 170 was detected as a possible lead with an increase in levels by 1057% (Table S1). Of note, this factor was shown to be required for efficient axonal regeneration [49], and phosphorylation at tyrosine 170 protects c-Jun from ubiquitination and degradation. [50]. 

Another neurogenesis-related target with more than a two-fold change in the levels of expression, following the treatment with norvaline, is nuclear factor NF-kappa-B (NF-κB) p105 subunit phosphorylated at S932. Phosphorylation at S932 leads to the processing of the p105 precursor and to the p50 subunit generation, which is a DNA binding subunit as well as an effector [51]. Of note, NF-κB signaling was shown to initiate the early differentiation of neural stem cells [52]. Moreover, activation of NF-κB signaling increases proliferation and reduces apoptosis of NPCs, whereas its inhibition promotes apoptosis of the neural progenitors.

It is worth mentioning that several targets disclosed by the assay demonstrated a substantial reduction in levels. Among them is cyclin-dependent protein-serine kinase 5 (Cdk5). This kinase is a priority lead with an 85% reduction in levels when phosphorylated at tyrosine 15 (Table S1). Cdk5 plays a critical role in the CNS during development, and its over-activation is linked to neurodegeneration following AD, Parkinson’s and Huntington’s diseases [53]. Accordingly, the targeting of Cdk5 activity has been suggested as a treatment strategy for a list of neurodegenerative diseases [54]. 

Special attention goes to the substantial reduction (by 78%) in mitogen-activated protein-serine kinase p38 (p38d MAPK) levels following the treatment with norvaline. This kinase has been shown to be activated via β-amyloid peptide and induce Tau protein hyperphosphorylation [55]. Here too, the inhibition of p38 MAPK is suggested as a potential strategy for the treatment of AD [56]. 

Another target that showed a substantial 65% decrease in levels is histone deacetylase 5 (HDAC5) phosphorylated at serine 498. HDAC5 regulates the homeostasis of histone acetylation, which plays a central role in various brain disorders. Its phosphorylation on serine residue 498 results in its nuclear export and consequent de-repression of downstream target genes [57]. The targeting of HDAC5 has previously been proposed as a promising therapy for AD [58], and several HDAC5 inhibitors have been suggested as efficient therapeutic agents [59]. Remarkably, HDAC4 phosphorylated at serine 246 demonstrated a substantial 48% reduction in levels following the treatment. Of note, the phosphorylation at S246 yields a functionally active form of this deacetylase [60], which plays an essential role in the brain physiology and neurodegenerative disorders pathogenesis [61]. Therefore, the development of HDAC4 inhibitors is a potential therapeutic direction in AD treatment as well [62]. 

## 3. Discussion

Adult neurogenesis is a complex physiological process that plays a crucial role in the maintenance of normal cognitive functions. This process consists of progenitor cell proliferation, newborn cell migration, and eventually, their maturation [40]. Newborn neurons incorporate into existing functional networks. They are identifiable via various labeling techniques. Dentate gyrus progenitor cells proliferate in the SGZ and migrate into the granular layer of the dentate gyrus. Then, they differentiate and become postmitotic cells with a different phenotype. The new neurons extend their axons to the hippocampal CA3 region and send dendrites to the molecular layer, which functionally integrates them into the hippocampal network [40].

It is worth highlighting that the resident hippocampal precursors are progenitor cells, which are capable of proliferation and multipotential differentiation, though, are incapable of self-renewal [63]. Thus, the extra-hippocampal stem cells generate progenitor cells, which then migrate to the dedicated neurogenic area (SGZ) and proliferate there to produce progeny that differentiate into a population of DCX-expressing newborn neurons. The immature neurons, which express both DCX and PSA-NCAM, decorate the thin lamina underlying the SGZ, and migrate into the granule cell layer [40] (Figure 1 and Figure 2). These cells undergo synaptic integration by sending extensive processes towards the molecular layer and CA3 area, and eventually become typical postmitotic cells. 

Adult neurogenesis is an extremely vulnerable process, which is prone to alterations under numerous physiological and pathological conditions. Several lines of evidence suggest a substantial impairment of neurogenesis in AD, which is one of the earliest pathological characteristics of the disease, and its manipulation has been pursued as a potential therapeutic strategy [64].

Various AD animal models show age-dependent neurogenesis deficiency. Decreased proliferation of the hippocampal progenitor cells has been demonstrated in APPswe/PS1dE9 transgenic mice [65]. Also, 3 × Tg mice are characterized by meaningfully impaired adult neurogenesis [3]. Our results prove a substantial, about four-fold, reduction of the NPC density in 3 × Tg mice compared to WT animals (Figure 1E). Moreover, the DCX-positive cells in 3 × Tg mice are deficient of characteristic processes and do not penetrate the granular layer (Figure 1C). Remarkably, various approaches are capable of inducing neurogenesis in adult rodents, including environmental enrichment and enhanced physical activity [66]. Furthermore, numerous studies report a reversal of the decline in neurogenesis in transgenic AD murine models, including 3 × Tg mice, as a corollary of different treatment strategies [67,68,69]. 

Growing empirical evidence indicates a unique role of NO in adult neurogenesis [70]. Accordingly, several agents have been successfully trialed with a rationale to increase brain NO levels, including arginine [71] and NO-donor supplementation [6]. In this study, we investigated the effects of a different NO-inducing approach upon adult neurogenesis. We utilized an arginase inhibitor, the non-proteinogenic amino acid norvaline, to promote adult neurogenesis in a murine model of AD. It is worth mentioning that several potent inhibitors are competent in modulating arginase function. They represent a promising group of medicines for the treatment of cardiovascular and metabolic diseases. However, the most effective inhibitors are relatively toxic substances with limited clinical applicability. 

Here, we assessed the neurogenesis rate by quantitatively evaluating the proliferation and differentiation of NPCs in the dentate gyrus SGZ. We applied several popular neuronal markers to characterize the different stages of neurogenesis by means of immunohistochemistry. 

Previously, we used an advanced proteomics assay to evidence a significant (by 43%) elevation in NCAM protein levels following norvaline treatment in 3 × Tg mice brains [15]. In the present study, we scrutinized the spatial patterns of PSA-NCAM hippocampal expression in relation to the treatment. Of note, PSA-NCAM-positive immature neurons have been shown to contribute to the early steps in adult hippocampal neurogenesis, such as proliferation and differentiation [72]. Consequently, PSA-NCAM is used as both a survival and a migration-associated neuronal marker [73]. This biomolecule is required for newly generated neuron survival in vitro [74] and in vivo [75]. Likewise, the role of PSA-NCAM in migration regulation and in the stimulation of newly generated neuron processes outgrowth has also been suggested [76]. Our methodology revealed a significant increase in the hippocampal granular layer PSA-NCAM positive surface area (Figure 2C) and intensity (Figure 2D) following the treatment, which implies improvement in the newborn neuron survival rate and accords with our previous results. It is noteworthy that PSA-NCAM positivity, in contrast to DCX, is a characteristic of a broad spectrum of cell populations in the adult brain. Besides the NPCs, immature granular neurons, and even mature cells express PSA-NCAM [77]. Therefore, these two markers characterize partially overlapping but different cell populations. 

It is worth emphasizing that other groups have proven the sensitivity of PSA-NCAM hippocampal levels in AD mice to various treatments and even experiences [78]. In WT animals, PSA-NCAM up-regulation correlates with hippocampal-dependent learning [79]. Therefore, this particular marker reliably indicates the efficacy of the treatment strategy applied and monitors the improvements in hippocampal-dependent function. 

In order to evaluate the treatment-associated changes in neuronal and dendritic density, we studied the brain expression patterns of MAP2. MAP2 belongs to a family of heat-stable microtubule-associated proteins, which are responsible for polymerization, stabilization, and dynamics of the microtubule neuronal networks. Accordingly, MAP2 is vital for maintaining neuronal architecture, internal cell organization, cell division, and neuronal morphogenesis [80]. Of note, the levels of MAP2 are significantly diminished in the brains of AD patients [81]. Moreover, in vitro studies have shown that Aβ oligomers induce a time-dependent degradation of MAP2 in murine primary cerebral neurons [80]. Another in vitro study demonstrated the neuroprotective effect of curcumin, which up-regulates MAP2 expression in human neuroblastoma cells treated with Aβ oligomers [82]. Therefore, MAP2 levels in AD brains are potentially treatment-sensitive and can reflect the treatment efficacy. 

It is noteworthy that 3 × Tg mice exhibit an early neuronal loss [83] along with a significant reduction in the hippocampal spine density [16]. Previously, we applied Golgi staining and observed a significant increase in hippocampal spine density following norvaline treatment [16]. Here, we assessed the effects of the treatment on the neuronal and dendritic density in 3 × Tg mice via quantitative immunohistochemistry with MAP2 antibody. Remarkably, the hippocampi of 3 × Tg mice treated with norvaline showed significantly greater MAP2 signal than that of 3 × Tg control mice (Figure 3). These findings point to norvaline rescuing effects on neuronal and dendritic loss, which characterizes the development of memory deficits in 3 × Tg mice, and are in accord with our previously published data [16]. 

We applied an advanced phospho-proteomics assay to study the biological pathways involved in the treatment-associated phenotype, and disclosed 48 targets with a substantial, more than 45%, change in the expression levels (Table S1). The primary metabolic pathway, with several target proteins involved, is MAPK/ERK pathway (Figure 6). Previously, we have shown that norvaline significantly increases the hippocampal levels of MAPK/ERK dual-specificity kinase 1 (MEK1) together with KRas GTPase [15]. Here we prove these findings with another assay and demonstrate a substantial, more than two-fold, norvaline-related escalation in the MEK1 expression levels. Activation of MEK1 is known to be accompanied by phosphorylation at serine 222 [84]. Remarkably, the phosphorylated MEK1 at serine 222 is the most apparent target with the treatment-associated increase of 1495% (Table S1), which points to a critical role of this pathway in the norvaline-associated phenotype. The ERK pathway possesses a pivotal role in integrating external signals from epidermal growth factor (EGF) and other factors into vital events promoting cell proliferation and growth. In molecular biology, it is among the most investigated signaling cascades by virtue of its central evolutionary-conserved function. Of note, EGF is capable of inducing differentiation of neuroglia into precursor cells and, eventually neurons; therefore, this pathway is also critical for neurogenesis. Moreover, the EGF receptor in the brain mediates the effects that are related to neuronal survival [85]. 

The expression of constitutively active MEK1 has been shown to increase astrocyte density in the adult mammal brain; however, MEK1 deletion results in a persistent failure of gliogenesis and blocks glial progenitors specification [86]. Moreover, MEK hyperactivation caused by phosphorylation at S218/S222 in radial progenitors leads to a marked increase in the production of cortical astrocyte precursors and mature astrocytes [86].

In a previous study, we demonstrated a significant treatment-related increase in the NOS3 protein levels [16]. NOS3 transcription regulation in the mammal brain is under precise control by the MAPK/ERK pathway. NOS3 transcription is initiated by various stimuli, which lead to the activation of phosphatidylinositol 3-kinase [87]. Of note, this kinase demonstrated a significant increase following the treatment in our recent study [15]. The downstream enzymes of the MAPK/ERK pathway (MEK1 and ERK) show substantial activation. Accordingly, we speculate that this mechanism is responsible for the observed elevation in NOS levels.

It is worth mentioning that several neurodegeneration-associated factors demonstrated a substantial decline in levels following the treatment with norvaline. Among them, p38 MAPK, Cdk5, and HDACs, which are promising targets for AD treatment, and their inhibitors, are under intensive clinical investigation [56,59].

AD is accompanied by widespread neuroinflammation, and is characterized by chronic microglial activation and overproduction of proinflammatory cytokines [88]. Recent reports have suggested that proinflammatory cytokines, especially tumor necrosis factor-α (TNFα), negatively regulate adult mammal neurogenesis [89], whereas anti-inflammatory cytokines exert the opposite effect [88]. In our previous works, we have shown a significant effect of norvaline treatment upon the rate of microglial activation [16], and the levels of TNFα [15] in the brains of 3 × Tg mice. Accordingly, we suggest a supporting effect of norvaline upon adult neurogenesis through the reduction of neuroinflammation. 

The small cytokine CCL11, which is produced by neurons in the brain, has been shown to be associated with immune response modulation and protection against neuroinflammation in rats [90]. More recent data strongly implicate the effect of CCL11 on mouse NPCs. Wang et al., (2017) utilized a rodent model of hypoxia-ischemia-induced brain damage to demonstrate that CCL11 promotes the migration and proliferation of NPCs [46]. Therefore, we reasoned that norvaline treatment would promote endogenous neurogenesis through neuroprotective factors such as anti-inflammatory cytokines, particularly CCL11. We tested our hypothesis by analyzing CCL11 mRNA expression in the hippocampi of 3 × Tg mice in relation to norvaline treatment and found a significant 79% treatment-associated increase. Accordingly, we speculate that CCL11 is at least partially responsible for the phenotype observed in the norvaline-treated mice. This relationship, however, is an assumption until proven by other assays, and further research is needed to shed light on the specific mechanisms of CCL11 induction by norvaline and its role in adult neurogenesis. 

PAX6 is an evolutionarily conserved transcription factor, which contributes to both embryonic and adult neurogenesis as a multifunctional regulator [91]. Maekawa et al. (2006) utilized a PAX6-deficient rat model to elucidate the essential role of PAX6 in the production and maintenance of early NPC in the postnatal hippocampal neurogenesis [92]. Moreover, patients with PAX6 mutations demonstrate phenotypes that match age-associated neurological disorders [93]. Animal models prove the progressive age-associated reduction in the PAX6 brain levels [94]. Accordingly, alterations in PAX6 expression during aging are responsible for age-associated compromised survival of neurons and glial cells, which leads to memory deficits [95].

In an elegant study, Maurya & Mishra (2017) demonstrated age-dependent alterations in PAX6 binding to the regulatory elements of genes involved in immunological surveillance and energy homeostasis [93]. Also, they evidenced an interaction of PAX6 with the Presenilin-1 regulatory element, which indicates its role in AD pathogenesis. Here, we demonstrated a treatment-associated increase in PAX6 protein levels (Figure 5), which further indicated norvaline promoting effects on neurogenesis in 3 × Tg mice. 

**Figure 7:**
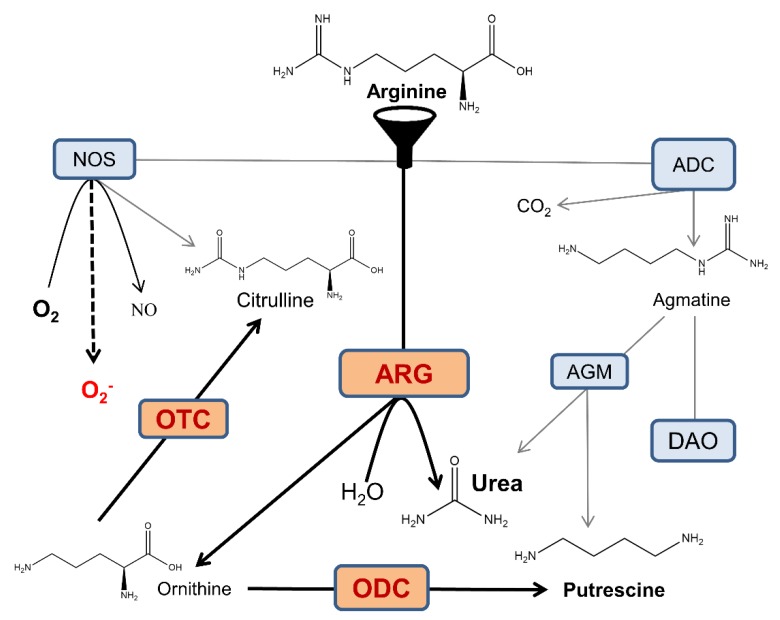
Crossroads of arginine metabolism in the AD brain. NOS oxidatively converts arginine into citrulline and NO. Arginase (ARG) hydrolyzes arginine into ornithine and urea. Arginine decarboxylase (ADC) produces agmatine and carbon dioxide via arginine decarboxylation. Agmatine is utilized in putrescine synthesis via agmatinase (AGM), and alternatively, the neurotransmitter GABA is synthesized in the diamine oxidase (DAO) pathway. Ornithine transcarbamylase (OTC) yields citrulline and phosphate. These pathways interfere with each other via intricate substrate competition mechanisms. For the sake of diagram simplicity, several intermediate steps and byproducts are omitted. In the AD brain, overactive arginase competes with NOS and ADC for the common substrate and reduces the bioavailability of arginine, which limits the production of agmatine and NO, and leads to NOS uncoupling and generation of superoxide anion. Overactivation of ornithine decarboxylase (ODC) leads to a surplus of downstream polyamine products, which can be neurotoxic [97]. Moreover, the gradual oxidation of polyamines by polyamine oxidase is associated with the generation of hydrogen peroxide and leads to oxidative stress [98].

Norvaline is an efficient non-competitive arginase inhibitor [96], and this feature is likely responsible for its neuroprotective properties. Of note, arginine is a common substrate for three enzymes present in several isoforms: arginase, NOS, and arginine decarboxylase (Figure 7). These enzymes compete for mutual substrate reserves; thus, the overactivation of any one of them leads to the deprivation of others. AD development is associated with arginase overexpression at sites of β-amyloid deposition [12,16], which leads to brain arginine deprivation and also NOS and arginine decarboxylase substrate deficiency. When NOS is deprived of arginine, it undergoes uncoupling, which leads to considerable alterations in its mode of function; these changes reduce the production of NO and generate superoxide anion, which in turn, leads to severe oxidative stress (Figure 7). 

NOS1 has been shown to be chiefly responsible for NO production in the brain and for regulation of vital physiological functions, including neurogenesis [11]. We demonstrated that norvaline upsurges the hippocampal levels of NOS1 [15] and NOS3 [16]; and therefore, increases the brain NO content. We speculate that the neuroprotective effects of norvaline are mainly mediated by NO generation and the reduction of oxidative stress, which is a principal characteristic of AD [99]. Moreover, NO has been shown to mediate neuronal differentiation of PC12 cells [100]. Thus, we suggest that the same mechanism is responsible for the phenotype observed in 3 × Tg mice treated with norvaline. 

The role of another arginine-processing enzyme in neurogenesis, arginine decarboxylase, which has been recently determined. Arginine decarboxylase is responsible for the conversion of arginine into agmatine [101]. Accordingly, arginase inhibition-associated improvement in the substrate bioavailability leads to an elevation in agmatine brain levels (Figure 7). 

Agmatine has been shown to increase the proliferation rate of cultured hippocampal rat NPCs in vitro in a dose-dependent manner and also to induce hippocampal neurogenesis in chronically stressed mice in vivo [102]. A more recent study in rats showed that agmatine attenuates traumatic brain injury consequences via promoting neurogenesis and the inhibition of gliosis [103]. Another group proved that agmatine regulates NPCs proliferation and their fate determination in the SVZ [104]. These authors applied immunoblotting and staining to show that agmatine increases MAP2 levels, which supports our findings. 

In summary, we have shown that long-term treatment with norvaline promotes NPC survival and differentiation in the hippocampi of 3 × Tg mice. Our study offers new insights into the controlling of NPCs function by manipulating the NO microenvironment in the brain. It also demonstrates arginase-targeting benefits in the treatment of AD. Our results provide compelling evidence pointing to norvaline as a multifunctional and potent agent, which is a promising candidate for further clinical investigations. The fact that the substance is a popular supplement with a long history of safe use by sportsmen makes it particular interesting candidate for the treatment of a wide variety of metabolic and neurodegenerative diseases.

## 4. Materials and Methods 

### 4.1. Animals and Treatment

Homozygous 3 × Tg mice, harboring PS1(M146V), APP(Swe), and tau(P301L) transgenes were purchased from Jackson Laboratory (Bar Harbor, ME, USA) and bred in our animal facility. These mice exhibit memory deficits associated with amyloid plaques deposition and tangle pathology [105]. 

Randomly chosen, male 4-month-old transgenic mice and age-matched male C57Bl/6 mice (wild-type) were divided into four groups (14 mice in each group) and housed in individually ventilated cages (Lab Products Inc., Seaford, DE, USA), with five mice per cage. The animals were provided with water and food ad libitum. The control animals received regular water. The experimental mice received water with dissolved (250 mg/L) norvaline (Sigma, St. Louis, MO, USA) for ten weeks in accordance with the previously published protocol [16]. The experiment lasted 2.5 months. The experiments were performed according to the “Guide for the Care and Use of Laboratory Animals” [106] and the experimental protocol (No 55-07-2018) was approved by Bar-Ilan University animal care and use committee. 

### 4.2. Tissue Preparation and Slicing

Four animals from each group were deeply anesthetized, with an intraperitoneal injection of 0.2 mL Pental (CTS Chemical Industries, Kiryat Malachi, Israel). The animals were perfused transcardially with ice-cold phosphate buffer saline (PBS), followed by ice-cold paraformaldehyde 4% in PBS. The mice were decapitated, and their brains were carefully removed and fixed in 4% paraformaldehyde for 24 h, and then were transferred to 70% ethanol at 4 °C for 48 h. The tissues were dehydrated and paraffin-embedded. The paraffin-embedded tissue blocks were chilled on ice and sliced on Leica RM2235 manual rotary microtome to a thickness of four µm. Then, the sections were mounted onto gelatin-coated slides, dried overnight at room temperature, and stored at 4 °C in slide storage boxes.

### 4.3. Quantitative Immunohistochemistry

We studied neurogenesis within the dentate gyrus of the adult mice hippocampal formation by means of immunohistochemistry. Staining was accomplished on the Leica Bond Max system (Leica Biosystems Newcastle Ltd., Newcastle upon Tyne, UK). Brain sections were dewaxed and pretreated with the epitope-retrieval solution (ER, Leica Biosystems Newcastle Ltd., Newcastle upon Tyne, UK), and then incubated for 30 min with primary antibodies. A Leica Refine-HRP kit (Leica Biosystems Newcastle Ltd., Newcastle upon Tyne, UK) served for hematoxylin counterstaining. The omission of the primary antibodies served as a negative control. 

Quantitative immunohistochemistry was accomplished using plane-matched coronal brain sections stained with appropriate antibodies, which produced a brown-colored end-product visible under a bright-field microscope. The coronal brain sections cut at 25 µm intervals throughout the brain per mouse (1.8–1.9 mm posterior to bregma) were used for the analysis. 

### 4.4. Doublecortin Labeling and Staining

Newly formed neurons were first labeled with doublecortin (DCX), whose expression is specific for newly generated neuronal cells [40]. We utilized the polyclonal antibody GTX134052 (GeneTex, Irvine, CA, USA) diluted at 1:500, to detect doublecortin protein, and quantified the number of DCX-positive neurons and the level of their stain intensity with Zen 2.5 software. The density of neural progenitors (DCX+ cells) in the dentate gyrus was calculated in a circle with a diameter of 385 µm^2^ and presented as the number of DCX+ cells per square mm. DCX+ objects with the surface area greater than 10 µm^2^ were taken into account. 

### 4.5. Polysialylated Neuronal Cell Adhesion Molecule Staining

Polysialic acid (PSA) is a homopolymer whose primary carrier in vertebrates is NCAM [107]. Commonly, DCX hippocampal expression is temporally in-frame with PSA-NCAM expression [40]. The molecule is exceedingly expressed in the brain during development; still, in the adult murine brain, newborn granule cells of the dentate gyrus highly express PSA-NCAM as well [108]. Accordingly, PSA-NCAM is a popular marker to study structural plasticity and neurogenesis in mammals.

In order to detect PSA-NCAM, we utilized the monoclonal antibody 12E3 #14-9118-82 (eBioscience™, Thermo Fisher Scientific, Waltham, MA, USA) diluted at 1:100. We quantified the PSA-NCAM-positive surface area and intensity within the dentate gyrus. Zen 2.5 with a preset threshold was used to measure these parameters in a circle with a diameter of 220 µm^2^.

### 4.6. Microtubule-Associated Protein 2 Staining

Microtubule-associated protein 2 (MAP2) is the most abundant brain MAP, which is predominantly expressed in dendrites and neuronal cell bodies during neurite outgrowth and dendritic branching [109]. 

The recombinant monoclonal antibody diluted at 1:20000 #ab183830 (Abcam, Cambridge, UK) was utilized for the detection of MAP2 protein. Bright-field micrographs of double-labeled sections have been used for quantitative analysis of immunohistochemistry. MAP2-positive objects with definite and unambiguous neuronal morphology within a circle with a diameter of 220 µm^2^ were analyzed. 

### 4.7. Imaging and Quantification

The brain sections have been viewed under an automated upright slide scanning microscope Axio Scan.Z1 (Zeiss, Oberkochen, Germany). The images were captured with 20 ×/ 0.8 and 40 ×/ 0.95 objectives at z-planes of 0.5 µm. An Axio Imager 2 Upright ApoTome Microscope was used to capture images with 100 ×/ 1.4 oil immersion objective.

Image analysis was carried out using ZEN Blue 2.5 (Zeiss). A fixed background intensity threshold was set for all sections representing a single type of staining. In order to create high-resolution data, the image deconvolution technique of entire z-series, with ZEN 2.5, was utilized. A computer-driven analysis was performed at each of the counting frame locations.

The surface of the immunoreactive area, above the preset threshold, was subjected to the analysis. The image densitometry method was applied to quantify the amount of staining in the specimens. The mean stain intensity of the specific channel was measured and presented as the average value for each treatment group.

### 4.8. Tissue Sampling, RNA Extraction, Reverse Transcription, and Real-Time Polymerase Chain Reaction

Five animals per group were rapidly decapitated with scissors. Their brains were carefully removed, and entire hippocampi were sampled. Total RNA was isolated from the left hippocampi using the RNeasy Mini Kit (Cat. No. 74104, QIAGEN, Hilden, Germany) following the manufacturer’s instructions including DNase treatment. RNA quantification was performed using Qubit™ RNA HS Assay Kit (Cat. No. Q32852, Invitrogen, Carlsbad, CA, USA). The RNA integrity was measured using Agilent 2100 Bioanalyzer System and Agilent RNA 6000 Pico Kit (Cat. No. 5067-1513, Agilent Technologies, Santa Clara, CA, USA). cDNA was prepared from 200 ng of total RNA using SuperScript^®^ III First-Strand Synthesis System for real-time polymerase chain reaction (RT-PCR) (Cat. No. 18080-051, Invitrogen) following the manufacturer’s instructions. RT-PCR was performed using TaqMan Ccl11: Mm00441238_m1 (Applied Biosystems, Foster City, CA, USA) probe. For the normalization of CCL11 RNA levels, ACTB endogenous housekeeping gene control ActB: Mm00607939_s1 was used. PCR was set in triplicates following the manufacturer’s instructions (Applied Biosystems, Insert PN 4444602 Rev. C) in a 10 μL volume using a five ng cDNA template. PCR was run, and the data was analyzed in the StepOnePlus system installed with StepOne Software v2.3 (Applied Biosystems, Foster City, California, USA). The quantification was performed using the comparative Ct (ΔΔCt) method [110].

### 4.9. Western Blotting

Hippocampal brain tissue from the right hemispheres was homogenized in lysis buffer (1% Triton, 10% glycerol, 120 mM NaCl, 25 mM HEPES, 1 mM EDTA, 0.75 mM MgCl_2_, 2 mM NaF, 1 mM Sodium vanadate, and Protease inhibitor). The samples were incubated on ice for ten minutes and then centrifuged at 11,000 g for 10 min at 4 °C to separate nuclei and residual tissue. Consequently, obtained supernatant was aliquoted and protein concentration was determined using a protein assay kit (BioRad, Hercules, CA, USA). Forty μg samples were subjected to sodium dodecyl sulfate-polyacrylamide gel electrophoresis and transferred onto a nitrocellulose membrane. The membrane was blocked for one hour at room temperature in TBS containing 0.1% casein. The membrane was incubated overnight with primary antibody 1:1000 dilution of Paired box protein (PAX6) #ab109233 (Abcam) and 1:1,000,000 dilutions of monoclonal anti-β-actin antibody produced in mouse #A5441 (Sigma). Following several washes with TBST, the membranes were incubated with LI-COR dye-conjugated secondary antibody for one hour. Membranes were then scanned on the LI-COR (LI-COR Biosciences, Lincoln, Nebraska, USA) Odyssey scanner.

### 4.10. Antibody Microarray

Five animals per group were rapidly decapitated and their brains were carefully removed. The entire hippocampi were carefully dissected, snap-frozen on dry ice, and stored at −80 °C.

The assessment of “hit” proteins’ expression was performed by the use of the Kinex KAM-1325 antibody microarray (Kinexus Bioinformatics, Vancouver, BC, Canada), in accordance with the manufacturer’s specification. The array features 875 phosphosite-specific antibodies (for phosphorylation) and 451 pan-specific antibodies (for expression levels of these phosphoproteins). The antibodies used on the microarray consist of polyclonal and monoclonal antibodies that were carefully selected and stringently validated in-house in over 25 different model systems. Each microarray consists of 2 identical fields, allowing two samples to be analyzed side by side at a time. Within each field, there are 16 subgrids of 13 × 13 spots. Diameters of spots average between 120 and 150 µm. 

The analyses were done with hippocampal lysates as described on the Kinexus web page (www.kinexus.ca). Briefly, pooled hippocampal lysates from five mice (each group) were labeled with biotin. Free biotin molecules were then removed after labeling reactions by gel filtration. An incubation unit was mounted onto the array with two samples (one control and one matching treated sample) side by side on the same chip. After the incubation procedure, unbound proteins were washed away and the array was probed with an anti-biotin antibody labeled with a fluorescent dye. Each array produced a pair of 16-bit images captured by a Perkin-Elmer ScanArray Reader laser array scanner (Waltham, MA, USA). Signal quantification was performed with ImaGene 9.0 from BioDiscovery (El Segundo, CA, USA) with predetermined settings for spot segmentation and background correction. The background-corrected raw intensity data were logarithmically transformed with base 2. Z scores were calculated by subtracting the overall average intensity of all spots within a sample from the raw intensity for each spot, and dividing it by the standard deviations of all of the measured intensities within each sample [111]. Z ratios were calculated by taking the difference between the averages of the observed protein Z scores and dividing by the SD of all of the differences for that particular comparison. In order to improve the analysis, the intensities of the signals were taken into account, and the changes in spot intensity between control and treatment samples were expressed as the percent change from control (%CFC) using globally normalized data. Globally normalized values for the signal intensity were colorized using the conditional formatting feature under the format tab in the pull-down menu of MS-Excel. 

### 4.11. KiNetscape Analysis and Representation

The key norvaline-induced changes in protein expression or phosphorylation of 45% or greater were extracted from the results of Kinex™ KAM-1325 antibody microarray analyses of the hippocampal lysates (Supplementary Table S1). For these protein target leads, the KinaseNET website database was queried to extract all known kinase-substrate interactions with these leads. In order to visualize the treatment-associated changes in the protein levels and/or phosphorylation, the data were imported into the Cytoscape 3.4 program (Institute For Systems Biology, Seattle, Washington, USA) and a KiNetscape protein kinase network map was generated.

### 4.12. Statistical Analysis

Statistical analysis was conducted with GraphPad Prism 8.3.0 for Windows (GraphPad Software, San Diego, CA, USA). The significance was set at 95% of confidence. The two-way ANOVA test was used to demonstrate whether the genotype, the treatment, or the interaction between both factors have an impact upon the phenotype. The two-tailed Student’s t-test was performed to compare the means of two groups. The Kolmogorov–Smirnov test served to evaluate the normality of the data distribution. All data are presented as mean values. Throughout the text and in plots, the variability is indicated by the standard error of the mean (SEM). 

## Supplementary Materials

The following are available online at https://www.mdpi.com/1422-0067/21/3/1133/s1.

## Abbreviations

AD	Alzheimer’s disease
NPCs	neuronal progenitor cells
3 × Tg	triple-transgenic mouse model of Alzheimer’s disease
NOS	nitric oxide synthase
Aβ	amyloid-beta
NFT	neurofibrillary tangles
SVZ	subventricular zone
APP	amyloid precursor protein
SGZ	subgranular zone
VEGF	vascular endothelial growth factor
BDNF	brain-derived neurotrophic factor
CNS	central nervous system
NRG	neuregulin
RT-PCR	real-time polymerase chain reaction
GDNF	glial cell-derived neurotrophic factor
RET	REarranged during Transfection
WT	wild-type
OD	optical density
ANOVA	analysis of variance
CA	cornus Ammonis
PSANCAM	polysialylated neuronal cell adhesion molecule
SEM	standard error of the mean
NGF	nerve growth factor
DCX	doublecortin
mRNA	messenger ribonucleic acid
PSD-95	postsynaptic density protein 95
MAP2	microtubule-associated protein 2
CCL11	C-C motif chemokine 11
PAX6	paired box protein 6
MAP	mitogen-activated protein
MEK1	dual specificity mitogen-activated protein kinase kinase 1
ERK	extracellular signal–regulated kinases
NF-κB	nuclear factor kappa-B
S	Serine
Cdk5	cyclin-dependent protein-serine kinase 5
p38dMAPK	mitogen-activated protein-serine kinase p38
HDAC	histone deacetylase
