# Supplementary material for: Arginase Inhibition Supports Survival and Differentiation of Neuronal Precursors in Adult Alzheimer’s Disease Mice
Source: Int J Mol Sci. 2020 Feb 8;21(3):1133. doi: 10.3390/ijms21031133 (PMC7037054; doi:10.3390/ijms21031133)
Supplement: Supplementary file 1 [file ijms-21-01133-s001.pdf]

Table 1. KAM-1325 array results. %CFC- the percent change of the treated sample in normalized intensity from the specified control. A %CFC value of 100% corresponds to a 2-fold increase in signal intensity with the treatment. A negative %CFC value indicates the degree of reduction in signal intensity from the selected control. The conditional formatting feature for %CFC was set to blue for -100%, and orange for +200%.

N	Target Name	P-Site	Full Target Protein Name	%CFC	Lead
1	MEK1	S222	MAPK/ERK protein-serine kinase 1 (MKK1)	1495	Possible
2	Jun (c-Jun)	Y170	Jun proto-oncogene-encoded AP1 transcription factor	1057	Possible
3	Src	Y419	Src oncogene-encoded protein-tyrosine kinase	924	Possible
4	Syk	Y323	Spleen protein-tyrosine kinase	799	Priority
5	MST1	T187	Mammalian STE20-like protein-serine kinase 1 (KRS2)	566	Possible
6	MEK1	T286	MAPK/ERK protein-serine kinase 1 (MKK1)	446	Possible
7	PDK1	Pan-specific	3-phosphoinositide-dependent protein-serine kinase 1	426	Priority
8	Wee1	Pan-specific	Wee1 protein-tyrosine kinase	422	Priority
9	Bmx (Etk)	Y40	Bone marrow X protein-tyrosine kinase	363	Possible
10	MST1	T183	Mammalian STE20-like protein-serine kinase 1 (KRS2)	360	Priority
11	GRK2	S670	Beta-adrenergic receptor kinase 1	334	Priority
12	TARDBP	S409+S410	TAR DNA-binding protein 43	285	Priority
13	NFKB1	S932	Nuclear factor NF-kappa-B p105 subunit	241	Possible
14	PFKFB3	S461	6-phosphofructo-2-kinase/fructose-2,6-biphosphatase 3	217	Priority
15	MEK1	Pan-specific	MAPK/ERK protein-serine kinase 1 (MKK1)	205	Priority
16	PTPN21	S637	Tyrosine-protein phosphatase non-receptor type 21	203	Possible
17	TH (TY3H)	S19	Tyrosine 3-monooxygenase	172	Priority
18	TRRAP	Pan-specific	Transformation/transcription domain-associated protein	167	Priority
19	p70S6K	Pan-specific	Ribosomal protein S6 kinase beta-1; Ribosomal protein S6 kinase 1	159	Possible
20	PRKACB	S339	cAMP-dependent protein-serine kinase catalytic subunit beta	156	Possible
21	ROCK2	Y722	Rho-associated protein kinase 2	150	Possible
22	Met	Y1003	Hepatocyte growth factor (HGF) receptor-tyrosine kinase	134	Priority
23	MAFG	S124	Transcription factor MafG	116	Priority

24	mTOR	S2478+S2481	Mammalian target of rapamycin (FRAP)	112	Possible
25	PDK1	S241	3-phosphoinositide-dependent protein-serine kinase 1	95	Priority
26	Frk	Pan-specific	Fyn-related kinase; Tyrosine-protein kinase FRK	95	Possible
27	NFkB p50	S337	Nuclear factor NF-kappa-B p105 subunit	94	Possible
28	PCYT1A	S329+S331	CTP: phosphocholine cytidylyltransferase isoform A	72	Possible
29	mTOR	Pan-specific	Mammalian target of rapamycin (FRAP)	66	Possible
30	STAG2	Y433	Putative uncharacterized protein DKFZp781H1753	60	Possible
31	PTEN	Pan-specific	Phosphatidylinositol-3,4,5-trisphosphate 3-phosphatase	55	Possible
32	GRK7	Pan-specific	Rhodopsin kinase	46	Possible
33	MKK3	S218	MAPK/ERK protein-serine kinase 3 beta isoform (MKK3 beta)	45	Possible
34	Kit	Y721	Mast/stem cell growth factor receptor protein-tyrosine Kit	45	Possible
35	CDK7	Pan-specific	Cyclin-dependent protein-serine kinase 7	-46	Possible
36	HDAC4	S246	Histone deacetylase 4	-48	Possible
37	CTNNB1	Y654	Catenin (cadherin-associated protein) beta 1	-55	Possible
38	PKD3	Pan-specific	Protein-serine kinase C nu; Serine/threonine-protein kinase D3	-64	Possible
39	HDAC5	S498	Histone deacetylase 5	-65	Possible
40	H2AFX	S139	Histone H2A.X	-74	Possible
41	IGF1R	Y1161+T1163	Insulin-like growth factor 1 receptor protein-tyrosine kinase	-74	Possible
42	p38d MAPK	Pan-specific	Mitogen-activated protein-serine kinase p38 delta	-78	Possible
43	CDK5	Y15	Cyclin-dependent protein-serine kinase 5	-85	Priority
44	Myc	S373	Myc proto-oncogene protein	-87	Possible
45	TEC	Y519	Tyrosine-protein kinase Tec	-88	Priority
46	JAK1	Y1022	Janus protein-tyrosine kinase 1	-88	Possible
47	RSK1(p90RSK)	S352	Ribosomal S6 protein-serine kinase 1	-90	Possible
48	RelB	Pan-specific	Transcription factor RelB	-91	Possible
